# Inflammatory Bowel Disease in Children: Current Diagnosis and Treatment Strategies

**DOI:** 10.7759/cureus.78462

**Published:** 2025-02-03

**Authors:** Akshita Bhalla, Anushka Shahi, Madhurima Maity, FNU Safa, Vindlacheruvu Srividya, Ruchira Clementina, Goutham R Anugu, Salma Younas

**Affiliations:** 1 Internal Medicine, Punjab Institute of Medical Sciences, Jalandhar, IND; 2 Internal Medicine, Sri Aurobindo Institute of Medical Sciences, Indore, IND; 3 Critical Care Medicine, Sir H.N Reliance Foundation Hospital, Mumbai, IND; 4 Internal Medicine, Osmania Medical College, Hyderabad, IND; 5 Internal Medicine, Sri Venkata Sai (SVS) Medical College, Hyderabad, IND; 6 Medicine, Government Medical College, Nizamabad, Nizamabad, IND; 7 Pharmacy, Punjab University College of Pharmacy, Lahore, PAK

**Keywords:** crohn’s disease (cd), inflammatory bowel disease (ibd), pediatric, treatment modalities, ulcerative colitis (uc)

## Abstract

Pediatric inflammatory bowel disease (PIBD), including Crohn’s disease and ulcerative colitis, has emerged as a significant global health challenge with rising incidence rates. Unlike adult inflammatory bowel disease, PIBD presents complexities, including growth impairment, nutritional deficiencies, and psychosocial challenges that necessitate tailored management strategies. This article reviews current diagnostic and emerging treatment strategies to highlight the evolution from traditional therapies such as aminosalicylates, corticosteroids, and immunomodulators to advanced biologic agents like infliximab and adalimumab. Emerging biological therapies, including vedolizumab and ustekinumab, show promise, while novel small molecule therapies such as Janus kinase (JAK) inhibitors are under investigation for potential use in the pediatric population. Supportive treatments, including exclusive enteral nutrition, modified diets, and probiotics, play a critical role in comprehensive disease management. Stem cell therapy and fecal microbiota transplant represent innovative approaches still under clinical evaluation. The review underscores the significance of holistic care, incorporating mind-body interventions and psychosocial support to improve patient quality of life. Key challenges persist, such as infection risks associated with long-term biological therapy use, gaps in pediatric-specific guidelines, and the limited inclusion of children in clinical trials. Future recommendations emphasize the importance of structured transition programs bridging pediatric and adult care, regular updates to clinical guidelines, and the integration of precision medicine to personalize treatment plans. Continued research and collaboration are essential for advancing the understanding and management of PIBD, ensuring that pediatric patients benefit from the most effective, evidence-based care available.

## Introduction and background

Pediatric inflammatory bowel disease (PIBD) is increasingly recognized as a significant global health concern, marked by rising incidence rates and complex treatment challenges. Inflammatory bowel disease (IBD), encompassing ulcerative colitis (UC) and Crohn’s disease (CD), is characterized by chronic inflammation of the gastrointestinal tract with alternating periods of relapse and remission. While UC is confined to the colon and results in mucosal ulceration, CD can affect any part of the gastrointestinal tract in a discontinuous manner [1,2]. Recent studies have reported a notable increase in the incidence and prevalence of PIBD, with some regions seeing a rise in pediatric cases by as much as 10-15% in the last decade [1,2].

The impact of PIBD extends beyond the physical symptoms, as children face unique challenges, including nutritional deficiencies, growth retardation, and psychosocial stress. These challenges contribute to a substantial disease burden and affect long-term development and quality of life [3-5]. Current data highlights that PIBD often leads to delays in both physical growth and social development, particularly in children diagnosed at an early age [3-5]. A 2016 report revealed that in the U.S., one in 1,299 children aged 2-17 was affected by IBD, showing an upward trend from 2009 [6]. Additionally, a study by Sýkora et al. has highlighted that Europe reports incidence rates as high as 23 cases per 100,000 person-years, while North America and parts of Asia report rates of 15.2 and 11.4 cases per 100,000 person-years, respectively [7]. A systematic review covering 131 studies from 48 countries between 2010 and 2020 found that North Europe and North America have the highest rates of PIBD, with 84% of studies indicating increasing trends [8].

PIBD diagnosis is particularly challenging due to the wide range of symptoms and the rapid disease progression observed in younger patients [9]. The EUROKIDS study reported that only half of unclassified IBD cases had a comprehensive diagnostic workup, although follow-up testing significantly reduced misclassification rates to 5.6% [10]. Early diagnosis is essential for effective management, as untreated pediatric CD can lead to complications such as strictures, internal fistulas, and growth impairment [11].

Biological therapies have revolutionized the management of PIBD by targeting specific immune pathways to achieve mucosal healing and sustained remission [12]. Recent studies on biologics have provided robust evidence of the effectiveness of anti-tumor necrosis factor (TNF) agents in improving long-term remission rates in pediatric patients, while also highlighting the risks of immunogenicity and loss of response over time. Anti-TNF agents like infliximab and adalimumab remain the only approved biologics for pediatric use, reserved for patients who do not respond to conventional treatments like exclusive enteral nutrition (EEN) or corticosteroids [13]. Combination therapies involving biologics and immunomodulators (e.g., methotrexate) have demonstrated better long-term outcomes in pediatric populations but require careful monitoring due to potential risks, including immunosuppression and increased susceptibility to infections. Combining biologics with immunomodulators has shown enhanced efficacy but poses the risk of immunogenicity and antibody formation [14]. Despite these advances, significant gaps in pediatric-specific treatment guidelines persist due to limited clinical trials.

Emerging therapies, including vedolizumab and ustekinumab, are under investigation, with early results showing promise. Ustekinumab has shown notable efficacy in patients with refractory CD, and vedolizumab's selective gut-targeting properties suggest it could provide an advantage over non-targeted agents like infliximab [15]. Meanwhile, novel approaches like fecal microbial transplantation (FMT) and stem cell therapy are gaining traction, especially for cases unresponsive to conventional treatments. The use of stem cell therapy is a promising option for patients with refractory disease, though safety and long-term effects remain a concern [16].

The increasing incidence of PIBD, coupled with its substantial impact on a child's physical and psychosocial development, highlights the importance of early diagnosis and a holistic treatment approach. This review aims to evaluate current diagnostic and therapeutic strategies for PIBD, identify gaps in existing pediatric guidelines, and propose areas for future research to enhance patient outcomes.

## Review

Significant advances have been made, particularly with biologics such as anti-TNF agents and emerging therapies; there remain considerable challenges in optimizing treatment for pediatric populations.

Risk factors of PIBD

The development of PIBD is influenced by a combination of genetic, environmental, and immune-mediated factors. Genetic predisposition plays a crucial role, with studies showing that first-degree relatives of IBD patients have a higher risk of developing the disease [17]. Specific genetic markers, such as mutations in the NOD2 gene, have been associated with a predisposition to CD [18]. Familial aggregation is also significant; up to 20% of pediatric patients have a family history of IBD, suggesting a hereditary component [19].

Environmental triggers include diet, antibiotic use, and changes in gut microbiota, which have been increasingly linked to the onset of IBD [20]. Children in urban areas show higher incidence rates compared to those in rural settings, highlighting the potential role of lifestyle and environmental exposures [20]. Early-life exposure to antibiotics has been associated with an increased risk of developing IBD, likely due to alterations in gut flora [21].

The increasing incidence of PIBD imposes a significant burden on healthcare systems worldwide. In 2019, there were 25,659 new cases and 88,829 prevalent cases of PIBD among children and adolescents globally, representing an increase of 22.8% and 18.5%, respectively, compared to 1990 [22]. This rise necessitates long-term, multidisciplinary management, including regular monitoring, medication, nutritional support, and psychological care. Consequently, healthcare systems face increased demands for specialized services, leading to higher healthcare utilization and associated costs. In the United States, the total annual healthcare costs for IBD were about $8.5 billion in 2018, with a significant portion attributed to pediatric cases [23]. The chronic nature of PIBD shows the need for comprehensive care strategies to manage the disease effectively and mitigate its impact on healthcare resources.

Beyond the physical toll, PIBD significantly impacts the psychosocial well-being of patients. Young patients often experience challenges related to growth failure, delayed puberty, and emotional and social difficulties due to the chronicity of their disease [24]. Studies have shown that PIBD can negatively affect school performance, social interactions, and overall quality of life [4]. These psychosocial impacts extend to the family, creating emotional and financial stress and necessitating psychological and social support.

Clinical presentation and diagnostic approaches

PIBD presents with a range of clinical manifestations that can vary widely, making early diagnosis challenging.

The most common gastrointestinal symptoms include persistent abdominal pain, chronic diarrhea (which may be bloody), and unexplained weight loss. These symptoms often reflect the extent and location of the inflammation in the gastrointestinal tract [25,26]. Beyond the digestive system, PIBD can present with extraintestinal manifestations that significantly impact the patient’s quality of life. These include joint pain or arthritis, skin conditions such as erythema nodosum or pyoderma gangrenosum, and growth failure due to malnutrition and chronic inflammation [27]. Growth delays are especially concerning children as they can lead to permanent stunting if not addressed [28]. 

The clinical presentation of PIBD can differ depending on the age at onset. In very early-onset IBD (diagnosed before age six), symptoms often include failure to thrive, delayed growth, and non-specific gastrointestinal complaints. These cases can present more extensive and severe disease, often requiring more aggressive diagnostic and treatment approaches [29]. Older children and adolescents typically present with more classic symptoms such as abdominal pain, diarrhea, and weight loss. They are more likely to report extraintestinal symptoms such as joint pain and may present with pubertal delays due to the chronic inflammatory state [1]. 

Accurate diagnosis of PIBD requires a comprehensive approach that combines clinical evaluation, laboratory testing, imaging, and histopathological confirmation. Initial diagnosis begins with a thorough clinical evaluation that includes a detailed patient history, physical examination, and assessment of growth parameters. Important historical details include the family history of IBD and previous episodes of similar symptoms [30,31].

Laboratory investigations are essential for supporting clinical diagnosis. Common tests include measurements of inflammatory markers such as C-reactive protein (CRP) and erythrocyte sedimentation rate (ESR). Anemia, elevated white blood cell count, and low albumin levels are also indicative of chronic inflammation [32]. Non-invasive imaging modalities, such as MRI and abdominal ultrasound, are used to evaluate the extent and severity of the disease. MRI is particularly useful for assessing small bowel involvement in Crohn’s disease [33]. Endoscopic evaluations, including colonoscopy and esophagogastroduodenoscopy, are key diagnostic tools that allow direct visualization of the gastrointestinal tract and enable biopsies to be taken for histopathological examination [34]. Biopsies obtained during endoscopic procedures are essential for confirming the diagnosis of IBD and differentiating between UC and CD [35]. Histopathological analysis can reveal specific features such as granulomas, which are indicative of CD [36].

PIBD can mimic various other conditions, making differential diagnosis crucial. Bacterial or viral infections present with similar symptoms of diarrhea and abdominal pain but typically have a shorter duration [1]. Chronic diarrhea and weight loss in children can also be seen in celiac disease, which is diagnosed through serological testing and confirmed by duodenal biopsy [37,38]. Food allergies and intolerances can mimic mild IBD symptoms, especially in younger children [39]. While IBS shares some overlapping symptoms such as abdominal pain and altered bowel habits, it lacks the inflammatory component present in IBD [40].

Screening Strategy for a High-Risk Pediatric Population

The screening strategy for PIBD is based on clinical red flag signs found in patients. The red flags indices are divided into major and minor signs [41]. The major red flag signs include overt blood in stools and/or perianal lesions like skin tags, hemorrhoids, fissures, fistulas, or abscesses. Minor red flag signs include a positive family history, unintentional loss in weight, and extra-intestinal manifestations of IBD like episcleritis, arthritis, and oral ulcers [42,43].

Positive serological tests include CRP levels of more than 10 mg/l, elevated ESR, and/or anemia. Fecal calprotectin is a novel biomarker essential for diagnosis of IBD. The advantage of measuring fecal calprotectin levels correlates to its sensitivity by excluding false negative results in patients with no major red flags [44,45].

**Figure 1:**
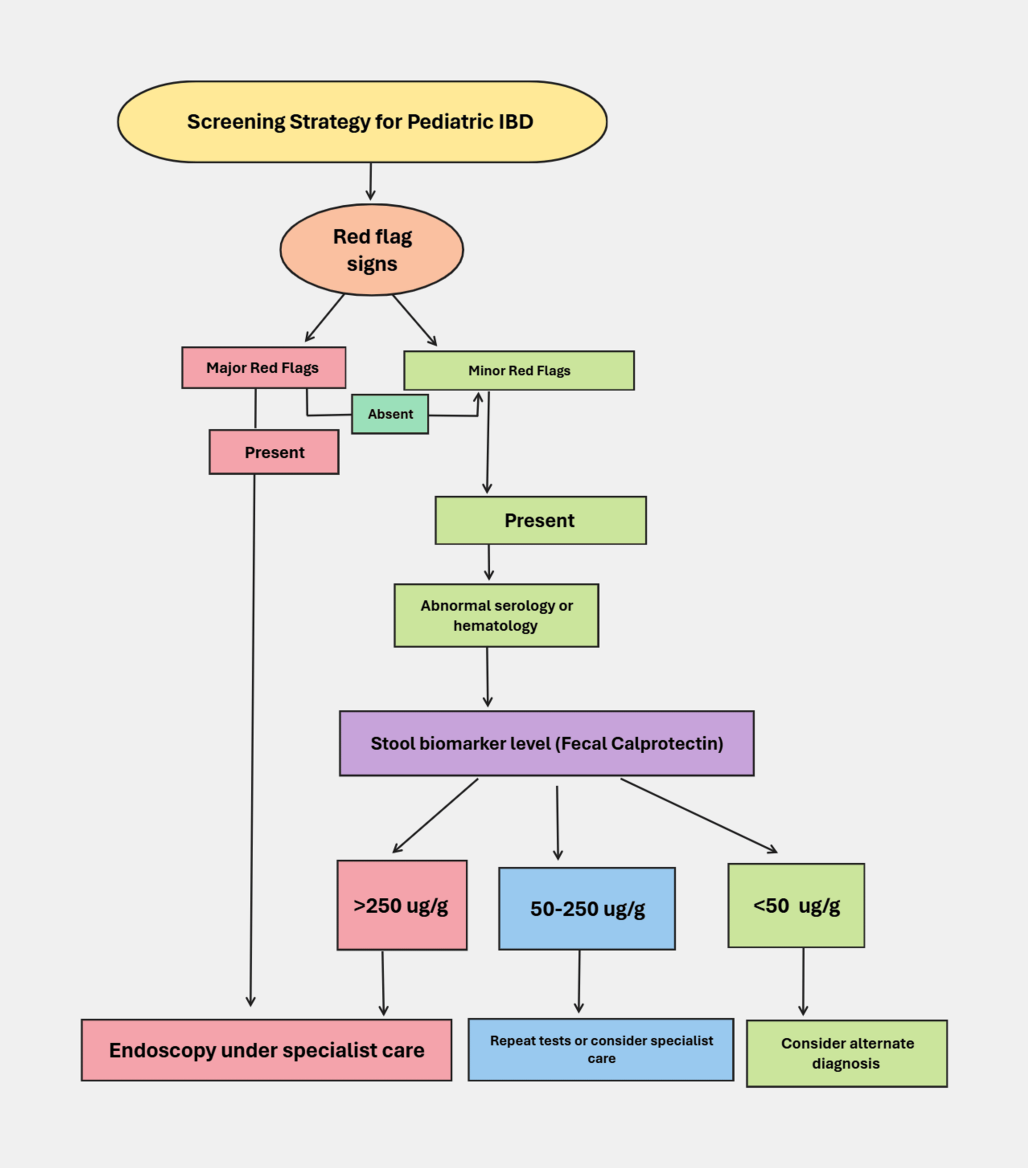
Screening strategy of PIBD PIBD: Pediatric inflammatory bowel disease

Figure 1 shows the flowchart of how the screening should be done for PIBD. This flowchart outlines the step-by-step process for screening pediatric patients for IBD, starting with the identification of red flag signs. Patients with major red flags are immediately recommended for endoscopy under specialist care. If minor red flags are present, further investigation is based on serology or hematology abnormalities and stool biomarker levels, particularly fecal calprotectin. Stool biomarker levels greater than 250 µg/g indicate the need for endoscopy under specialist care, while values between 50 and 250 µg/g may warrant repeated tests or referral to specialists. If levels are below 50 µg/g, an alternative diagnosis should be considered. This strategy ensures a systematic approach to diagnosing PIBD, prioritizing early detection and appropriate specialist involvement.

Early diagnosis and timely initiation of treatment also help in reducing morbidity associated with disease in the long run. This can be achieved by raising awareness among the parents about the red flag signs which they need to remain vigilant about. These include the major red flags like overt rectal blood loss or the minor ones like family history of IBD, unintended weight loss, or other atypical extra intestinal manifestations of PIBD [41]. 

Apart from this, it is also crucial that parents be properly counseled regarding their child’s condition and given psychosocial support if necessary, as IBD in a child affects both the child and the family, an important consideration often overlooked [4]. 

Diagnostic delays are the major contributing factor to the poor prognosis of the disease in children due to lack of awareness among parents [46].

**Figure 2:**
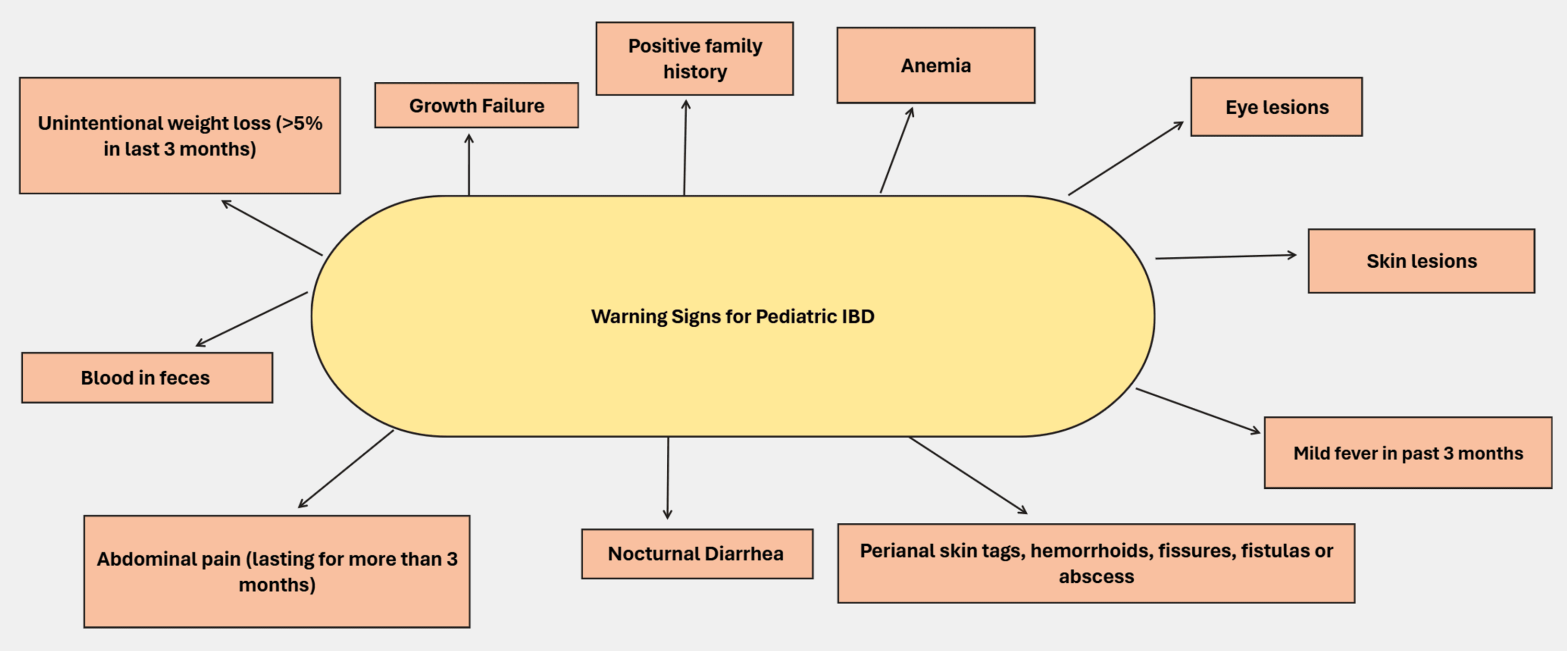
Warning signs of PIBD PIBD: Pediatric inflammatory bowel disease

Figure 2 shows the warning signs of PIBD. This flowchart highlights the warning signs that may indicate the presence of PIBD. It includes a range of gastrointestinal and extra-intestinal symptoms, such as abdominal pain, unintentional weight loss, and blood in stools, which are commonly observed in PIBD cases. Additional red flags like growth failure, anemia, perianal skin tags, eye lesions, and skin lesions further raise suspicion for PIBD. Children with any combination of these warning signs should be evaluated promptly, with a focus on early diagnosis and appropriate management. The presence of multiple warning signs warrants further investigation, potentially involving laboratory tests and imaging studies as outlined in the screening strategy.

Current biological agents

Current biological agents offer an alternative treatment for PIBD when conventional therapies fail [47]. While traditional treatments such as aminosalicylates, corticosteroids, and immunomodulators are often employed as initial strategies, they have limitations, especially in moderate-to-severe cases [48]. These limitations include adverse side effects with long-term use and reduced efficacy in addressing the underlying disease pathology over time. Consequently, biologic therapies have gained prominence due to their targeted approach and ability to achieve sustained remission and mucosal healing (MH) [48].

Infliximab (IFX) and adalimumab (ADA) are currently the primary first-line biologics approved for PIBD. Other biologics like golimumab, vedolizumab, ustekinumab, and tofacitinib are in different phases of clinical trials in pediatric patients [49]. The choice of biologic therapy depends on the patient’s treatment history, disease severity, and clinical response to prior treatments. IFX and ADA are anti-TNF agents that have demonstrated efficacy in inducing and maintaining remission in both CD and UC when conventional treatments do not yield sufficient results [50]. IFX is generally the first-line biologic, especially for patients with moderate-to-severe disease who are unresponsive to or intolerant of step-up therapies like corticosteroids or aminosalicylates. IFX, a chimeric monoclonal antibody, was the first biologic approved for pediatric use and is indicated for patients who do not respond to standard therapies, as well as for those with perianal fistulizing CD [51]. ADA, a fully human monoclonal antibody, serves as an effective alternative, particularly when patients develop a loss of response or intolerance to IFX [52]. In PIBD, ADA is typically used after IFX failure or when there is a loss of response to IFX, offering an alternative for ongoing disease control. Both agents have shown that when used appropriately, they can improve clinical and endoscopic outcomes, ultimately reducing hospitalization and the need for surgical interventions [50-52].

Certolizumab pegol (CZP), while less commonly used, is another biologic agent for the treatment of moderate-to-severe CD and is approved in certain regions, including the USA, Switzerland, and Russia. CZP’s primary advantage over IFX and ADA lies in its higher efficacy in neutralizing TNF-α and its lower potential for inducing ADAs, which makes it a preferred option in some cases where reducing immunogenicity is a priority [53]. However, CZP is still limited in its pediatric approval and usage and thus not as widely utilized as IFX or ADA.

The combination of biologics with immunomodulators, such as methotrexate, is another strategy that has been explored to enhance treatment efficacy and reduce the formation of ADAs. This approach has shown that combining biologics with immunomodulators can prolong the effectiveness of biologics by decreasing immunogenicity. However, it is not without risk; the combined immunosuppressive effect raises concerns about increased susceptibility to infections and the potential for rare but severe complications like hepatosplenic T-cell lymphoma, particularly in male patients [49].

The biologic therapy approach is expanding, and a comprehensive understanding of available options is critical. The following table summarizes the current biologics used for PIBD, including their mechanisms of action, indications, side effects, and pediatric approval status (Table 1).

**Table 1:** Summary of current biologics used for PIBD PIBD: Pediatric inflammatory bowel disease

Drug	Mechanism of Action	Indications	Adverse Effects	Contraindications	Dosage and Duration of Treatment	Pediatric Approval
Infliximab [51]	Anti-TNFα monoclonal antibody	Moderate-to-severe CD and UC; perianal CD	Infusion reactions, immunosuppression, psoriasis, lymphoma	Multiple sclerosis, CHF, active tuberculosis; hepatitis B and hepatitis C; HIV infections; hypersensitivity to murine products.	Intravenous dosage of 5mg/kg at 0, 2, and 6 and 8 weeks or intravenous dosage of 10 mg/kg at every 4-6 weeks.	Yes
Adalimumab [52]	Fully human anti-TNFα monoclonal antibody	CD unresponsive to infliximab; UC	Injection site reactions, infections, rare malignancies	Multiple sclerosis, CHF, active tuberculosis.	For induction: 2.4 mg/kg subcutaneously (maximum 160 mg) at 0 weeks 1.2 mg/kg (maximum 80 mg) at week 2. For maintenance: 0.6 mg/kg every other week.	Yes
Vedolizumab [53]	Anti-α4β7 integrin monoclonal antibody	Moderate-to-severe CD and UC	Infections, potential risk of PML, hypersensitivity	Severe Infections, hypersensitivity, progressive multifocal leukoencephalopathy (PML)	For induction: intravenous 300 mg with 0, 2, 6 weeks. For maintenance: 300 mg every eight weeks	Under pediatric trials
Ustekinumab [54]	Anti-IL-12/23 monoclonal antibody	CD and UC	Infections, infusion reactions, skin issues	Hypersensitivity, active infections, live vaccines	Intravenous 6 mg/kg, [maximum 520 mg].	Under pediatric trials
Certolizumab pegol [55]	PEGylated anti-TNFα monoclonal antibody	Moderate-to-severe CD	Lupus-like syndrome, rare T-cell lymphoma	Hypersensitivity reactions to certolizumab	100 mg to 800 mg subcutaneously every two to four weeks for maintenance of remission.	Limited pediatric use

Infliximab (IFX) 

It is the first biological agent that is approved by the FDA for CD. IFX (trade name Remicade) is a chimeric IgG1 monoclonal antibody against TNF-α having 75% human and 25% murine sequences. Indications for IFX usage in PIBD include unresponsive or intolerant to step-up treatment, including corticosteroids, aminosalicylates, and immunomodulators [56]. Induction therapy for perianal fistulizing disease. IFX is a potent agent in reducing the likelihood of hospitalization by half and surgery by 33-77% [57].

In a study conducted on pediatric CD patients naïve to biological agents, clinical (Pediatric Crohn's Disease Activity Index (PCDAI)) and endoscopic (Simple Endoscopic Score for Crohn's Disease (SES-CD)) evaluations performed at time 0 and followed up after 9-12 months. Results showed that biologics improve mucosal lesions and seemingly better so if given in combination with immunomodulators [58]. Simultaneous usage of immunomodulators with biologicals reduces the rate of antibody production although there is an increased risk of developing hepatosplenic T-cell lymphoma, especially in male pediatric CD patients. Side effects of IFX include mainly immunosuppression, which leads to opportunistic infections like tuberculosis and hepatitis. Others include infusion reactions like injection site erythema and swelling. Psoriasiform and cutaneous reactions are also common. IFX is typically associated with the development of Lupus-like syndrome, which presents as classical drug-induced lupus [59,60].

Adalimumab

It is the second anti-TNFα monoclonal antibody that is approved for pediatric patients. Unlike IFX, it is fully humanized. It is the treatment of choice for CD after IFX failure and in patients who have become unresponsive to IFX during the treatment duration. There are increased remission rates in patients with moderate to severe CD in relation to serum ADA concentration, as shown by the study of Wyneski et al. [61]. In the study conducted by Fumery et al., results concluded that ADA is safe and effective in two-thirds of patients with pediatric-onset CD and IFX failure [62]. The side effects of ADA are namely immunosuppression, psoriasiform lesions, and rarely lymphoma. In similarity with IFX, ADA also leads to clinical as well as endoscopic remission of CD in terms of MH [52]. 

Certolizumab Pegol (CZP)

It is an anti‐TNFα agent for the treatment of moderate to severe CD. CZP is approved for CD primarily in the USA, Switzerland, and Russia only. The advantage of using CZP over IFX and ADA is that CZP was able to neutralize TNFα with higher efficacy by increasing regulatory T-cell expression. Along with that, there was less antidrug antibody production with CZP in comparison to IFX and ADA [63,64].

Current guidelines

Current guidelines from both the European Crohn’s and Colitis Organization (ECCO) and the European Society for Paediatric Gastroenterology, Hepatology, and Nutrition (ESPGHAN) emphasize early intervention with biologics for children at risk of severe disease or those who do not achieve remission with exclusive enteral nutrition or corticosteroids. ECCO-ESPGHAN guidelines also recommend considering anti-TNF therapy for maintenance and induction of remission in patients with delayed growth or severe disease presentations [65]. North American guidelines, provided by the North American Society for Pediatric Gastroenterology, Hepatology and Nutrition (NASPGHAN), echo these recommendations but also highlight the importance of combination therapy to enhance treatment durability and reduce the risk of antibody formation against biologics [66].

Therapeutic drug monitoring (TDM) is highlighted as a crucial component of effective biologic therapy management in PIBD. TDM involves regular measurement of drug levels and anti-drug antibodies (ADAs) to tailor dosage adjustments, ensuring optimal drug exposure and maintaining clinical remission. This approach can mitigate secondary loss of response and guide decision-making when switching therapies or adjusting doses [67].

There are considerable gaps in the guidelines regarding the pros and cons of combination therapy in PIBD due to a lack of adequate drug studies in the pediatric population. Most biological agents undergo extensive adult testing before being considered for pediatric use, leading to a delay in their approval for younger patients [47]. While combination therapy is shown to be beneficial, the long-term safety profile, especially concerning risks like infection and rare malignancies, remains an area of concern. The availability of new biologics, such as vedolizumab and ustekinumab, holds promise, but further pediatric-focused research and updated clinical guidelines are needed to solidify their place in treatment protocols [68].

Emerging biologic agents in PIBD

Vedolizumab 

Vedolizumab (VDZ) is a humanized gut-selective anti-integrin monoclonal immunoglobulin G1 antibody that has been FDA-approved (GEMINI 1 & 2) for induction and maintenance therapies in adult IBD (UC and CD). It specifically targets the α4β7 heterodimer, a surface marker of intestinal lymphocytes and thus decreases T-lymphocyte migration to the inflamed intestinal tissues and thereby reducing the inflammatory load of IBD. Due to its gut-specific molecular action, it doesn’t interfere with lymphocyte migration to other systemic tissues, unlike its predecessor natalizumab which had to be withdrawn due to its lymphocytic inhibitory actions on brain tissues resulting in progressive multifocal leukoencephalopathy [69]. Its clinical response and remission rates in adult CD at six weeks were reported as 31.4% & 14.5% respectively and in adult UC response rate at six weeks was 47.1% compared to their placebos [70]. Currently, its use in PIBD is still off-label even though it has been recommended for use in children as second- or third-like step-up drug in resistant cases of UC & CD by European Society for Pediatric Gastroenterology Hepatology and Nutrition (ESPGHAN) and European Crohn's and Colitis Organization (ECCO) since its independent clinical trials in the pediatric population are still pending [71]. VDZ’s ongoing phase 3 trial for PIBD is expected for completion by 2024 [72].

Ustekinumab 

Ustekinumab (UST) is a humanized IgG1k monoclonal antibody that blocks interleukin activations (IL-12 & IL-23) and subsequent differentiation of T-cells (Th1 cells) involved in inflammatory response by binding to their p40 subunit and inhibiting their receptor interactions. Its clinical efficacy for induction and maintenance remission in adult IBDs has been proven and FDA-approved from multiple studies (CERTIFI, UNITI, and UNIFI) with best responses from treatment-naïve patients. Its concurrent use in other autoimmune conditions (psoriasis, arthritis) makes it a valuable biologic agent for evaluation in PIBD [67]. Its post-induction remission rates in adults remained comparable in CD (34%) & UC (39%) with a safe and acceptable adverse effect profile [73]. Currently, UST is undergoing phase 3 clinical trials for PIBD with an expected completion by 2025 [74]. In a few studies conducted in small groups of children, UST has shown higher efficacy in CD than UC with a significantly improved PCDAI, especially in anti-TNF-resistant patients [75].

Tofacitinib 

Tofacitinib (TOF) is a pan-Janus kinase (JAK-1 & -3) inhibitor that hinders the JAK pathway and associated intracellular signaling, thus downregulating the inflammatory cytokines and growth factors contributing to IBD pathogenesis. Inspired by its efficiency in rheumatic arthritis (RA), TOF was subsequently investigated as a new agent in IBD due to similar autoimmune inflammatory pathologies of RA and IBD. Post the phase III randomized control trial, TOF has found acceptance as a biologic agent in adult UC (moderate-severe) only with subpar outcomes in CD (OCTAVE 1 & 2). Even though efficacious in adults, its safety profile is still under scrutiny with herpes zoster and pulmonary embolism being the severe adverse effects recorded thus far in patients treated with TOF daily [76]. In a single-center study conducted on 21 children and young adults, TOF showed promising results in UC and IC patients with substantial clinical improvement in 43% of subjects at the end of the 12-week induction [39]. TOF’s ongoing phase 3 trial for PIBD is expected for completion by 2026 [77].

Other Agents 

Newer biologics such as mirikizumab and risankizumab are under exploration as potential treatments for PIBD. Mirikizumab, an IL-23p19 antagonist, has shown promise in adult CD and UC by selectively inhibiting the IL-23 pathway, which is implicated in chronic inflammation [78]. Risankizumab, another IL-23p19 inhibitor, has already shown efficacy in adult CD clinical trials and is being considered for pediatric studies. Although these agents provide hope for targeted, less immunogenic treatments, more pediatric-specific trials are necessary before their routine use can be recommended [79].

Despite advances in biologic treatments for PIBD, there remain significant challenges. Pediatric drug trials are limited by logistical and ethical constraints, which often result in a lag between adult and pediatric approvals. The smaller patient population further complicates large-scale studies, impacting the availability of robust data to guide treatment. Safety profiles for long-term use in children remain unclear for many newer biologics.

Non-biological and supportive treatments

Aminosalicylates (Sulfasalazine and Mesalamine)

The oldest class of targeted drugs, 5-aminosalicylic acids, are oral therapies that are efficacious and safe for mild to moderate UC induction and maintenance. Oral or rectal 5-ASA maintains UC remission better than placebo. Oral and rectal 5-ASA work similarly or better in distal UC. The combination of oral and intermittent rectal 5-ASA appears to be more beneficial. Alternatives include formulations that provide considerable 5-ASA levels in the distal colon [80]. Due to life-threatening side effects such as Stevens-Johnson syndrome and subacute hepatic failure, 5-ASA without sulfa was developed. It is sometimes given to youngsters who cannot take medications as a liquid [81]. For pediatric UC, corticosteroids, aminosalicylates, or both induce remission, and maintenance treatment is aminosalicylate monotherapy (in Europe, often with a thiopurine) [82].

Corticosteroids 

UC and CD treatment begins with corticosteroids. Corticosteroids improve inflammation management but not intestinal repair. Corticosteroids induce remission but can create systemic side effects such as hypertension, hyperglycemia, and bone issues, making them a long-term treatment [82]. Oral or rectally administered budesonide has considerable liver first-pass metabolism, minimizing systemic corticosteroid side effects.

*Calcineurin Inhibitors* 

Methotrexate is the immunomodulator of choice in North America to maintain remission by decreasing drug antibody development [83]. Thiopurines (azathioprine or 6-mercaptopurine) were once widely recommended, but clinicians now hesitate [84]. Long-term thiopurine use can cause rare but dangerous reactions such as lymphoma and non-melanoma skin cancer. Thiopurines are rarely used in North America saved for autoimmune liver disease. Thiopurines were traditionally popular in Europe, but currently, they are used individually [83]. Biological drugs are the main treatment for CD and severe UC.

Antibiotics

IBD treatment includes antibiotics for intra-abdominal abscesses, perirectal disease, fistula formation, infectious gastroenteritis, dysbiosis modulation, and short-term adjunctive therapy. Use for small-bowel CD and acute UC or CD colitis is controversial due to insufficient clinical trials [75]. Current IBD therapy options underutilize antibiotics, which directly target bacteria and microbiome. Antibiotics can affect IBD by lowering luminal bacteria numbers and possibly promoting beneficial bacteria, preventing bacterial tissue invasion and translocation [85]. Randomized controlled trials in adults have examined antibiotic therapy for IBD; however, the findings are inconsistent and no recommendations can be given [86]. Breton et al. reported the largest retrospective cohort of 63 children with moderate to severely active IBD-colitis (CD 42.8%, UC 36.5%, IBD-U 20.6%), unresponsive to steroids or biological failure [87].

A combination of 3-5 oral antibiotics (amoxicillin, doxycycline, metronidazole, ciprofloxacin, vancomycin, the “Jerusalem cocktail”) caused a clinical response in 65.3% of children and remission in 39.7%. This population may benefit from combination therapy for rescue or steroid sparing. Antibiotic-responsive children also reacted well to a second course of treatment for a disease flare [85]. Different oral amoxicillin, metronidazole, doxycycline, and vancomycin combinations reduced PUCAI scores in 7/8 patients and even remitted 3 of them. AZCRO and PRASCO are well-designed multicenter trials that indicate antibiotic efficacy but not SOC therapy or equivalent efficacy [81,88]. Antibiotic side effects, antibiotic resistance, and gut microbiota effects make antibiotic use less appealing and less feasible [89].

Emerging Small Molecule Therapies

Recent advances in the field of small molecule therapies have focused on targeted oral agents that inhibit specific intracellular signaling pathways involved in IBD pathogenesis. JAK inhibitors, such as tofacitinib, have been approved for adult UC and are being evaluated for pediatric use. These inhibitors work by blocking the JAK-STAT pathway, which mediates cytokine signaling involved in immune activation. While effective, tofacitinib and other JAK inhibitors come with safety concerns, including increased risk of infections and thromboembolic events [81].

Other emerging small molecules include TYK2 inhibitors and S1P receptor modulators like ozanimod, which are being explored for their potential in treating IBD. Early-phase clinical trials have demonstrated promising results in terms of reducing disease activity and maintaining remission, but long-term data on safety and efficacy in pediatric populations are still lacking. These therapies, if proven safe and effective, could offer more convenient, non-invasive treatment options that may be integrated into current treatment regimens or used as adjuncts to biologics [76].

Probiotics

ISAPP defines probiotics as “live microorganisms that, when administered in adequate amounts, confer a health benefit to the host” [90]. Many probiotics come from healthy people's commensal gut microbiota and try to replace the dysbiotic microbiome that fails to regulate gut inflammation. They resemble “healthy” gut bacteria and create homeostasis. Safety makes probiotics popular among adult and PIBD patients. The treatment seems more “natural” to patients [91].

Stem Cell Therapy

Current treatments struggle to achieve permanent remission and MH. Stem cells (SCs) can modify immunity, decrease inflammation, and have anti-apoptotic and pro-angiogenic actions, making them an ideal treatment for chronic inflammation and intestinal damage in IBD. HSCs and MSCs have been found to treat IBD in recent years. In addition, many clinical investigations have examined MSCs' disease-treating efficacy [92].

The therapeutic objective of autologous HSCT would be to restore the primary immune system of the patient (resetting of the immune system), after using chemotherapy to eliminate self-reactive T lymphocytes (lymphoablation) and memory cells, which would constitute the effectors of the immune dysregulation observed in CD, thereby inducing antigen tolerance over the long term.

Nutritional and Dietary Interventions 

Dietary treatments have historically played a crucial role in the treatment of IBD in children. Children with IBD are susceptible to malnutrition and may need nutritional replenishment. During a time when corticosteroids and 5-aminosalicylic acids were the sole medicinal treatments available, the use of specific formula diets became a fundamental approach for addressing nutritional deficiencies in patients with IBD. Additionally, these diets were frequently used to alleviate symptoms associated with consuming conventional diets [81]. The correlation between diets consisting solely of formula and the healing of the bowel has led to the exploration of dietary manipulation as a key method of treatment [93]. 

The notion of EEN originated and became a key therapeutic approach for pediatric Crohn's disease in Europe and has been moderately adopted in the United States. The precise processes underlying the observed therapeutic responses remain unknown; however, initial research has suggested that changes in the intestinal flora may play a role [94]. The restricted practicality of a diet consisting solely of formulas has resulted in the development of modified enteral regimens that combine diet with partial enteral nutrition. Additionally, elimination diets are being investigated as potential therapy options. The specific carbohydrate diet, CD exclusion diet, and Mediterranean diet all incorporate strategies to decrease the consumption of dietary additives, animal products, and refined grains, while promoting the intake of fruits and vegetables [95,96].

The precise therapeutic benefits of diet have yet to be adequately defined. Implementing a nutritional strategy when medical treatment is the most effective option is difficult, partly due to the lack of comprehensive clinical research on dietary therapy. Both the medical team and the kid and family must carefully evaluate the risk of illness progression while waiting for any therapy to reach its maximal effectiveness. Successful implementation of dietary therapy requires a competent and committed team, which includes a specialized dietitian for IBD [97]. 

The IBD dietician, like the IBD behavioral health professional, needs to have knowledge of the indications, manifestations, complexities, and therapies associated with IBD. Parents are exposed to significant alternative nutritional regimens through the Internet and well-intentioned contacts. These may encompass unfamiliar dietary supplements and potentially dangerous mixtures. Parents may withhold this information from their treating physicians but frequently choose to disclose it to an IBD-registered dietician whom they have confidence in. The effectiveness of exclusive enteral nutrition in CD can also be achieved by following an exclusion diet that eliminates processed foods. This approach may help address the issue of limited palatability associated with liquid formula [98].

Gut Microbiota Modulation

Modification of the intestinal flora is still expected as a treatment strategy because of the long-established role of changed microbial composition to the onset and persistence of IBD. Aiming to directly restore a dysbiotic microbiome, FMT involves delivering donor stool to the patient's intestinal lumen and has shown success in adult UC patients. It is now a well-established course of treatment for children with IBD who have recurrent Clostridium difficile [99,100]. Still being researched, however, is the potential of FMT for IBD in the absence of C. difficile; certain adult UC trials have shown promise. Its effectiveness in children with CD and UC is still being studied [101]. According to a newly published paper, parents and children taking part in these trials find FMT therapy from anonymous, screened donors to be acceptable and tolerable [102].

The therapeutic practice of introducing healthy fecal bacteria into patients is known as FMT. The goal of FMT is to replenish the gut microbiota with a healthy bacterial population in order to restore the colonic microbiota. Three primary areas are addressed by FMT techniques: donor selection, donor substance preparation, and FMT delivery. For lower gastrointestinal delivery, the stool must weigh at least 25 g in each of the FMT preparations, and for upper gastrointestinal delivery, it must weigh at least 12.5 g [103]. According to Halaweish et al. (2022), the final fecal substance preserved frozen at a temperature of −80°C is thought to have a maximum shelf life of two years. Before being used, the frozen substance is to be thawed at 37 degrees for six hours [104]. A study found that the most common short-term adverse events of FMT included abdominal discomfort, flatulence, abdominal distension, borborygmi, and low-grade fever. The long-term adverse events include arthritis/arthralgia, urticaria, depression, allergic bronchitis, and partial sensorineural hearing loss. In addition to re-establishing the immune system and preserving the equilibrium of intestinal microecology, FMT can enhance the diversity of intestinal microorganisms. The final outcome may depend on a number of factors, including the donor's characteristics, the host genotype, the disease's progression, the use of antibiotics associated with the onset of the illness, and the particular types of dysbiosis linked to IBD. Because it is a novel treatment for IBD, its safety and efficacy are still unknown, and patient acceptability is low. As a result, longer-term follow-up studies are necessary [105]. There must also be requirements for donor selection standards. Better techniques and more efficient protocols to prepare fecal substances and administer FMT will be realized through additional research.

Combination biologic therapies 

The concept of using combination biologic therapies is a novel and evolving approach aimed at enhancing treatment efficacy for patients who do not respond adequately to monotherapy. Combining different classes of biologics, such as anti-TNF agents with anti-integrin or anti-IL agents, targets various pathways in the inflammatory cascade, potentially yielding more comprehensive disease control [106]. Early case reports and observational studies have shown that this approach can lead to improved outcomes in treatment-refractory PIBD cases. For example, the combined use of ustekinumab (an IL-12/23 inhibitor) with VDZ (an anti-integrin agent) has been explored with some success, although the safety profile and risk of immunosuppression require further examination [107]. Larger, controlled studies are needed to establish efficacy, safety, and best practices for using combination biologic therapies in PIBD.

Combination biologic therapies offer promise for refractory PIBD but come with significant risks. High costs could limit accessibility, particularly in resource-limited settings. Combining agents increases immunosuppressive risks, raising concerns about infections and malignancies, especially in pediatric patients with developing immune systems. The long-term safety of these therapies is still unclear, with potential impacts on growth and development.

While promising, these therapies require more research through controlled trials to assess safety, efficacy, and the optimal patient profile. A cautious, evidence-based approach is necessary until more data are available.

Challenges in managing PIBD

One of the significant challenges in managing PIBD is the issue of immunogenicity associated with the long-term use of biologic therapies. Immunogenicity refers to the development of ADAs that neutralize the biologic agent's effectiveness, resulting in a loss of response over time [108]. This phenomenon is particularly prevalent with anti-TNF therapies like IFX and ADA, where up to 30% of patients can develop ADAs. Loss of response can lead to disease relapse, increased inflammation, and the need for dose escalation or switching to alternative treatments, which complicates the management of PIBD. To mitigate these issues, combination therapy with immunomodulators like methotrexate is often recommended, although this approach carries its own set of risks, including heightened immunosuppression [108].

TDM has become an essential tool for optimizing the use of biologic agents in PIBD. TDM involves measuring drug concentrations and ADA levels to tailor dosing and maximize treatment efficacy [109]. Proactive TDM measuring drug levels at scheduled intervals even when the patient is asymptomatic can help maintain appropriate drug exposure and prevent the development of ADAs [110]. Reactive TDM, on the other hand, is used in response to clinical symptoms or loss of response to treatment. Implementing TDM allows clinicians to make informed decisions about adjusting doses, switching therapies, or adding immunomodulators [111]. While TDM has been shown to improve outcomes, standard protocols for its implementation are still evolving, and the practice is not uniformly applied across all treatment centers [112].

PIBD has a significant psychosocial impact not only on the child but also on their family. The chronic and unpredictable nature of IBD can lead to emotional distress, anxiety, and depression in young patients [113]. Children may face challenges related to self-esteem, social interactions, and school performance due to frequent absences and physical symptoms. The condition's impact extends to the family, who often experience increased stress and financial burden [114]. 

Despite advances in the treatment of PIBD, there remain substantial gaps in clinical guidelines. Current guidelines for PIBD often borrow heavily from adult treatment protocols due to the limited number of pediatric-specific studies. This leads to variability in treatment practices and creates challenges in standardizing care.

Developing more comprehensive, evidence-based pediatric-specific guidelines and conducting larger-scale pediatric trials will be essential to bridge these gaps. Continued research and collaboration among global PIBD networks are crucial to ensuring that guidelines evolve alongside advancements in treatment and clinical practice.

Cost of IBD care in children

The financial burden of PIBD is a significant concern, especially given the increasing incidence of the disease and the complex treatment needs of affected children. 

A study by Fondell et al. (2019) quantified the first-year healthcare costs for children newly diagnosed with IBD. Among the 67 patients analyzed, the mean cost of care in the first year post diagnosis was $45,753. For children with CD, the cost was $43,095, while those with UC had higher mean costs at $50,516. Severe cases of IBD were associated with significantly higher costs; severe CD patients had a mean cost of $71,176, and severe UC patients had a cost of $134,178. These higher costs were primarily driven by the need for more intensive therapies, including biologic infusions. Infusion therapy, a critical component of treatment for moderate to severe IBD, accounted for a large portion of these costs. Children receiving infusion therapy had a mean cost of $59,376, compared to $27,903 for those who did not require infusions. The cost distribution showed that 37% of total expenses came from infusion therapy, 25% from hospital costs, and 18% from outpatient procedures. Costs related to outpatient oral medications, imaging, and visits accounted for smaller portions, highlighting the diverse factors contributing to the overall healthcare expenditure [115].

The study emphasizes the need to consider these cost components when planning for the management of PIBD, especially in severe cases, where the financial burden can be particularly high. The growing use of biologics for refractory cases of IBD also plays a significant role in these escalating costs, underscoring the importance of cost-effective treatment strategies.

Future recommendations and research directions

The transition from pediatric to adult IBD care is a critical phase that requires a structured approach to ensure continuous and effective disease management. Future research should focus on multicenter pediatric trials to evaluate the effectiveness of transition programs. These programs should emphasize education, self-management, and coordinated care, aiming to reduce flare-ups and improve long-term outcomes. In addition, research should explore strategies for optimizing the transition process, such as developing standardized communication protocols between pediatric and adult care teams.

To keep pace with emerging treatments, PIBD treatment guidelines need to be regularly updated to incorporate the latest therapies. Research should also investigate adherence-monitoring systems to ensure consistent implementation of guidelines and improve patient outcomes. Precision medicine holds great potential in personalizing PIBD treatment by integrating genetic, environmental, and microbiome factors. However, future studies must focus on the long-term safety of emerging therapies, especially in pediatric populations. Key areas of research include multicenter trials that focus on safety, efficacy, and dosing in children, as well as long-term studies to assess the lasting impact of therapies on growth and development. Finally, including pediatric patients in clinical trials is essential to advancing PIBD treatment. It is important to prioritize studies that evaluate the long-term safety and optimal therapeutic strategies for children, ensuring that therapies are specifically tailored to their unique needs.

## Conclusions

The management of PIBD has made significant strides, with advances in biologic therapies, emerging non-biologic interventions, and supportive treatments playing key roles in improving patient outcomes. Despite these developments, challenges remain, particularly in managing long-term treatment efficacy, addressing immunogenicity, minimizing infection risks, and providing comprehensive psychosocial support. The need for better, pediatric-specific guidelines and more inclusive clinical trials underscores the ongoing challenges in PIBD care.

Findings from current research and evolving treatment strategies suggest that clinicians should adopt a more holistic approach to managing PIBD, incorporating not only pharmacological treatments but also supportive care and psychosocial interventions. Proactive use of therapeutic drug monitoring, combined with patient education and family support, can enhance treatment adherence and overall outcomes. Implementing structured transition programs can ensure continuity of care as patients move into adult treatment settings, addressing a significant gap in current practice.
